# Wall-thickness-dependent strength of nanotubular ZnO

**DOI:** 10.1038/s41598-017-04696-4

**Published:** 2017-06-28

**Authors:** Na-Ri Kang, Young-Cheon Kim, Hansol Jeon, Seong Keun Kim, Jae-il Jang, Heung Nam Han, Ju-Young Kim

**Affiliations:** 1 School of Materials Science and Engineering, UNIST (Ulsan National Institute of Science and Technology), Ulsan, 44919 Republic of Korea; 2 Center for Electronic Materials, KIST (Korea Institute of Science and Technology), Seoul, 02792 Republic of Korea; 3 Division of Materials Science and Engineering, Hanyang University, Seoul, 04763 Republic of Korea; 4 School of Materials Science and Engineering, Seoul National University, Seoul, 08826 Republic of Korea; 5 KIST-UNIST Ulsan Center for Convergent Materials, UNIST, Ulsan, 44919 Republic of Korea

## Abstract

We fabricate nanotubular ZnO with wall thickness of 45, 92, 123 nm using nanoporous gold (np-Au) with ligament diameter at necks of 1.43 μm as sacrificial template. Through micro-tensile and micro-compressive testing of nanotubular ZnO structures, we find that the exponent *m* in $\bar{\sigma }\propto {\bar{\rho }}^{m}$, where $\bar{\sigma }$ is the relative strength and $\bar{\rho }$ is the relative density, for tension is 1.09 and for compression is 0.63. Both exponents are lower than the value of 1.5 in the Gibson-Ashby model that describes the relation between relative strength and relative density where the strength of constituent material is independent of external size, which indicates that strength of constituent ZnO increases as wall thickness decreases. We find, based on hole-nanoindentation and glazing incidence X-ray diffraction, that this wall-thickness-dependent strength of nanotubular ZnO is not caused by strengthening of constituent ZnO by size reduction at the nanoscale. Finite element analysis suggests that the wall-thickness-dependent strength of nanotubular ZnO originates from nanotubular structures formed on ligaments of np-Au.

## Introduction

Functional metal oxides such as ZnO have been widely used for sensor, energy conversion, and catalyst materials^1–7^. Because of its excellent piezoelectric properties, ZnO has been widely studied for applications in nano-generators that convert mechanical energy to electrical energy, and various ZnO structures have been suggested to achieve high conversion efficiency and mechanical reliability^8–10^. Nano-accordion structures of ZnO and Al-doped ZnO have been fabricated by using interference lithography and atomic layer deposition (ALD), and its stretchability attains 51%, i.e. two orders of magnitude greater than the planar film structure^8^. ZnO nano-sheet structures proposed as nano-generators provide highly enhanced stretchability by using elastic buckling of the structure with high aspect ratio^9, 10^. The reliability of these devices relies on the mechanical properties of bulk structure and constituent materials.

Recently, three-dimensional micro-architectures with superior mechanical properties have been suggested^11–17^. Meza *et al*. fabricated three-dimensional nano-structural metamaterials by ALD coating of metal oxide on a polymer nano-lattice patterned by direct laser writing followed by selective etching of the polymer template using oxygen plasma etching^14^. They are ultralight and so energy-absorbent that they can recover their original shape after compression to greater than 50% strain. During compression in the thin-walled structure, brittle fracture in the constituent solid can be suppressed by optimizing the wall thickness-to-tube radius ratio, resulting in ductile-like behavior by dominant elastic shell buckling. Various hollow nano-lattices with low density and high elastic deformation limit have been suggested, and the relation between the structure of the nano-lattice and wall thickness has been studied. Schaedler *et al*. suggested metal micro-lattices prepared by electroless plating of Ni on three-dimensional polymer templates; these materials show complete recovery after compression to greater than 50% strain, and degradation in compressive strength is lower than 10% after repeatable compressions^15^. Biener *et al*. introduced nanotubular TiO_2_ and Al_2_O_3_ with ultralow density^16^. They synthesized ultralow-density bulk materials with interconnected nanotubular morphology using nanoporous gold (np-Au) as template. These nanotubular materials provide superior mechanical properties in terms of hardness-to-weight ratio, as investigated by nanoindentation. These three-dimensional micro-architectures with hollow and/or lattice structures have excellent mechanical performance such as high energy absorption and high strength-to-weight ratio, overcoming brittle nature of constituent materials.

Here we fabricate nanotubular ZnO using ALD on np-Au followed by selective etching of the np-Au template, and perform micro-tensile and micro-compressive testing. We compare tensile and compressive behavior of nanotubular ZnO structure. The strength of the constituent ZnO, as evaluated by the Gibson-Ashby model looking at the strength of a cellular structure as a function of relative density, is found to increase with decreasing wall thickness. We propose that the wall-thickness-dependent strength of nanotubular ZnO comes from material or structural properties and investigate this proposition with hole-nanoindentation, grazing incidence X-ray diffraction (XRD), and finite element analysis (FEA).

## Results and Discussion

### Tensile and compressive behavior of nanotubular ZnO

**Figure 1:**
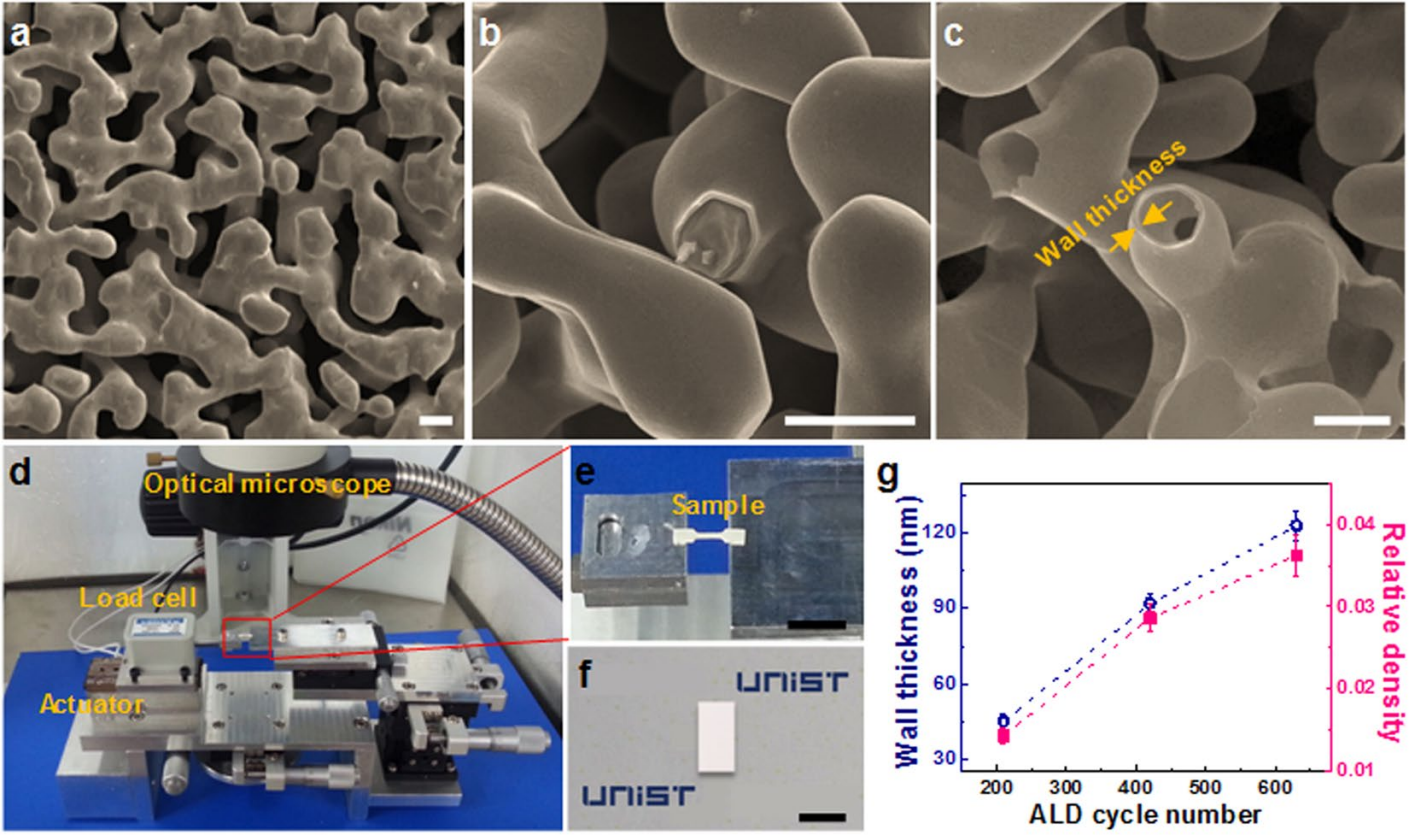
Nanotubular ZnO for micro-tensile and micro-compressive testing. (**a**) Sacrificial nanoporous gold (np-Au) with average ligament size 1.43 μm (scale bar, 2 μm). (**b**) ZnO wall coated on ligaments of np-Au using ALD (scale bar, 2 μm), and (**c**) nanotubular ZnO prepared by selective etching of np-Au template (scale bar, 2 μm). (**d**) Custom-built *in-situ* micro-tensile tester equipped with optical microscope (OM), (**e**) nanotubular ZnO gripped in micro-tensile tester (scale bar, 5 mm) and (**f**) shape of micro-compressive sample (scale bar, 1 mm). (**g**) Wall thickness and relative density as a function of ALD cycle number.

**Figure 2:**
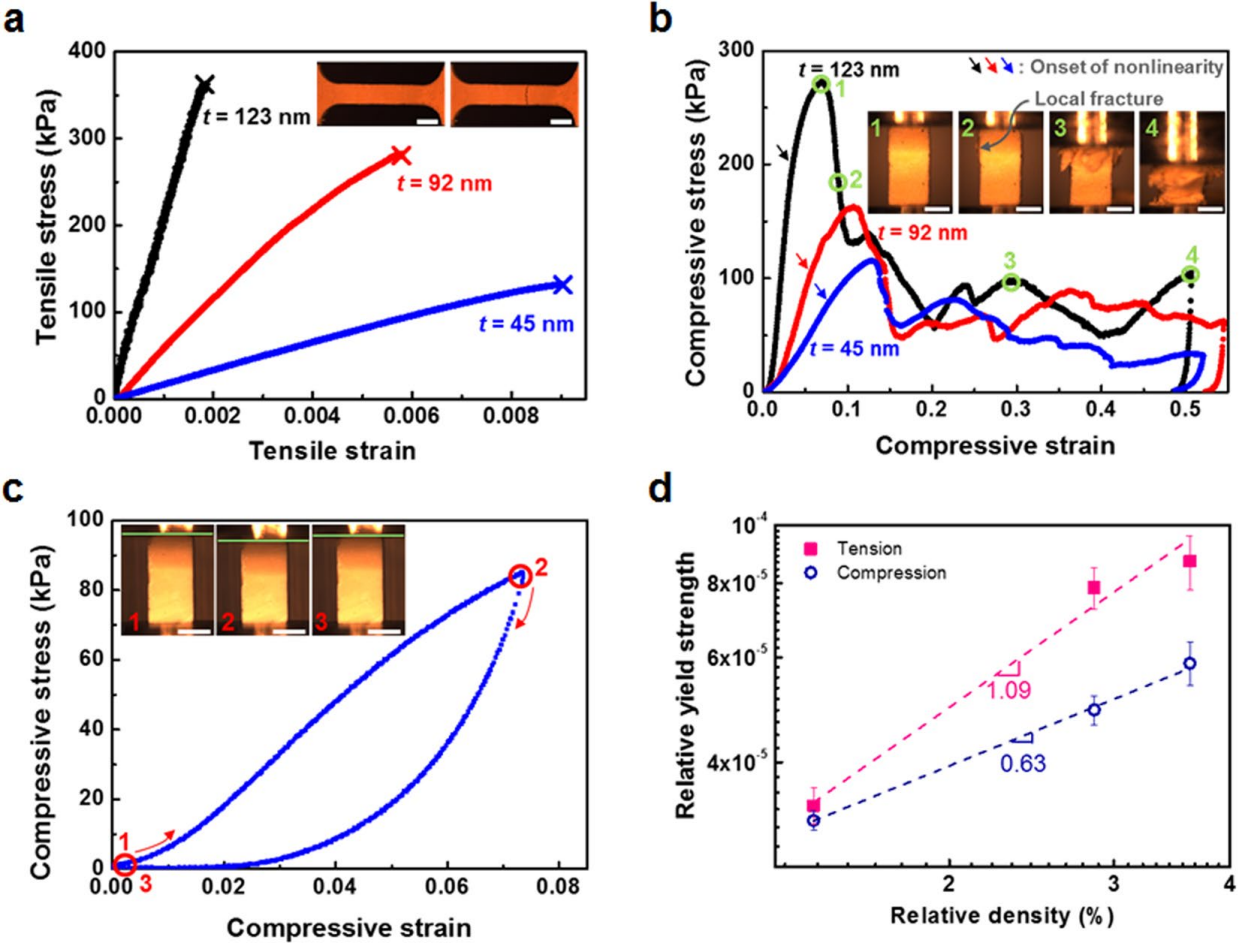
(**a**) Tensile behavior of nanotubular ZnO. Inset: OM images before and after micro-tensile testing (scale bars, 500 μm). (**b**) Compressive behavior of nanotubular ZnO. Insets: OM images of compressed nanotubular ZnO at points 1 through 4 in compressive stress-strain curve for *t* = 123 nm (scale bars, 500 μm). (**c**) Interrupted unloading testing for *t* = 45 nm. Insets: OM images of nanotubular ZnO at points 1 through 3 in stress-strain curve (scale bars, 500 μm). (**d**) Relative yield strength, ratio of yield strength of nanotubular ZnO structure to strength of constituent ZnO measured by hole-nanoindentation, 3.9 GPa as a function of relative density.

Nanotubular micro-tensile and micro-compressive ZnO samples with wall thickness, *t*, of 45 (±3), 92 (±4), and 123 (±6) nm are shown in Fig. 1 (see Methods and Supplementary Fig. S1). Hereafter, these nanotubular ZnO samples with wall thickness of 45, 92, and 123 nm are referred to as *t* = 45, 92, 123 nm, respectively. We measured the volume and weight of bulk nanotubular ZnO samples at millimeter scale, from which relative density, ratio of volume occupied by constituent solid to total volume of nanotubular ZnO, was measured with density of solid ZnO, 5.61 g/cm^3^. As shown in Fig. 1g, relative density is 1.4% (±0.10%) for *t* = 45 nm, 2.8% (±0.16%) for *t* = 92 nm, and 3.5% (±0.24%) for *t* = 123 nm. Figure 2a shows typical tensile stress-strain curves for nanotubular ZnO, indicating brittle behavior in tension, linear elastic deformation and catastrophic failure (see Supplementary Movie 1 for *t* = 123 nm). Fracture strain is 0.91% (±0.05%) for *t* = 45 nm, 0.61% (±0.06%) for *t* = 92 nm, and 0.18% (±0.01%) for *t* = 123 nm. They show negligible plasticity in tension, so these fracture strains are almost identical to tensile elastic limit of nanotubular ZnO samples. Measurement of the tensile elastic limit is critical since this property determines the critical bending radius of flexible devices and maximum tensile strains of stretchable device where nanotubular materials are used. Compared with fracture strain of 0.03% for bulk ZnO^18^, tensile elastic limits of nanotubular ZnO structures are greatly improved values. Fracture strength is 131 (±10) kPa for *t* = 45 nm, 289 (±7) kPa for *t* = 92 nm, and 363 (±8) kPa for *t* = 123 nm. All nanotubular ZnO samples were made with identical np-Au templates, and hence the nanotubular structures are identical and differences in mechanical behavior probably come from different wall thicknesses of constituent ZnO.

Figure 2b shows typical compressive stress-strain curves for *t* = 45, 92, 123 nm. They show similar trends as compressive strain increases regardless of wall thickness: stage (i) linear elastic deformation, (ii) nonlinear increase in stress between onset of nonlinearity and highest stress, followed by (iii) gradual decrease in stress, and (iv) wavy plateau (see Supplementary Movie 2 for *t* = 123 nm). The onset of nonlinearity as indicated by arrows in Fig. 2b, boundary between stages (i) and (ii), is attributed to elastic shell buckling and/or local fracture. As shown in Fig. 2c, we performed interrupted unloading during stage (ii) for *t* = 45 nm. Unloading curve shows anelastic loop-type recovery rather than reversible elastic recovery, and the sample does not recover its original shape; instead, about 2% plastic deformation remains after unloading. This indicates that during stage (ii), elastically recoverable shell buckling and local fracture leading to plastic deformation occur simultaneously. The critical ratio of wall thickness *t* to tube radius *a* for elastic shell buckling of straight tubes in three-dimensional architectures^14, 19^ is given by

1 $${(\frac{t}{a})}_{crit}=\frac{{\sigma }_{f}}{E}\sqrt{3(1-{\nu }^{2})},$$

where *σ*_*f*_, *E*, *v* are respectively the fracture strength, elastic modulus, and Poisson’s ratio of the constituent solid. The critical ratio of wall thickness to radius for nanotubular ZnO is estimated to be 0.063 for *t* = 45 nm, 0.128 for *t* = 92 nm, and 0.172 for *t* = 123 nm where a tube radius of 715 nm, the average radius of ligament necks in np-Au, is used. The right-hand term in Equation (1) is 0.0859 when *σ*_*f*_ = 3.9 GPa, *E* = 77 GPa, and *v* = 0.2^20, 21^ are used. *σ*_*fs*_ and *E* are measured by hole-nanoindentation, as described below. The critical ratio in *t*he left term for *t* = 45 nm is smaller than right term, 0.0859 and left terms are greater than right term for o*t*her two samples, *t* = 92 nm and *t* = 123 nm. If the nanotubular ZnO is composed of long and straight tubes^14^, this may mean that the *t* = 45 nm sample could show ductile-like behavior by dominant shell buckling while the *t*=92 nm and *t*=123 nm samples could show brittle-like behavior by dominant local fracture. However, no transition in deformation mode depending on wall thickness was observed, and the three samples show similar behavior in tension and compression. Unlike three-dimensional architectures composed of long, straight, and hollow struts, the nanotubular ZnO in this study was formed on np-Au ligaments with mostly saddle-shape and convex-shape surfaces^22^: this results in curved shells, np-Au ligament diameter at necks that is thinnest inner diameter of tubes is somewhat distributed, connectivity between ligaments is irregular, and tubes are randomly distributed^23, 24^. Due to these structural features, a mixed mode of elastic shell buckling and local fracture is likely to operate widely simultaneously after onset of nonlinearity.

Compressive yield strain at boundary between stages (i) and (ii), is 7.5% (±1.9%) for *t* = 45 nm, 5.3% (±0.7%) for *t* = 92 nm, and 3.8% (±1.0%) for *t* = 123 nm. These values are approximately one order of magnitude greater than the elastic limits in tension. During stage (iii), local fractures are observed widely, as indicated in Fig. 2b inset. Locally-fractured shells can hardly support external force until densification stage at extremely high strain, suggesting that any decrease in stress is caused by extensive local fractures. In previous reports on compression of hollow-tube lattice of NiP with relative densities ranging from 0.01% to 0.54%^15^, hollow-tube lattice structures with high relative density showing brittle-like behavior by dominant local fracture have a stress-drop region similar to ours while those with low relative density do not. One feature of the stress drop in stage (iii) is that discrete strain bursts are not observed, implying that fine local fractures occur gradually. Stage (iv) (Fig. 2b inset) shows severe collapse of the nanotubular ZnO structure.

Figure 2d shows the relation between relative yield strength, ratio of yield strength of nanotubular ZnO structure to fracture strength of the constituent solid, (3.9 GPa is used for the fracture strength of the constituent solid, ZnO nanotubular walls, which is measured by hole-nanoindentation as described below), and relative density in log-log scales. The linear slope for tension is found to be 1.09 and that for compression is 0.63. By Gibson and Ashby^25^, the ratio of brittle-crushing strength for open-cell foams with a box-like central tubular void size *t*_*i*_ within struts of thickness *t*_*s*_ to the fracture strength of the constituent solid is

2 $$\frac{{\sigma }_{cr}}{{\sigma }_{f}}=C\frac{1+{(\frac{{t}_{i}}{{t}_{s}})}^{2}}{\sqrt{1-{(\frac{{t}_{i}}{{t}_{s}})}^{2}}}{\bar{\rho }}^{1.5},$$

where *C* is the proportional constant. This equation suggests that linear slope is 1.5 in Fig. 2d if the fracture strength of the constituent solid is independent of wall thickness. Linear slopes in Fig. 2d, 1.09 in tension and 0.63 in compression, are smaller than 1.5 as in Equation (2), which means that the fracture strength of the constituent solid increases as wall thickness decreases. In other words, if yield strengths of nanotubular ZnO structures in tension and compression in Fig. 2d are converted to fracture strength of constituent ZnO as a function of wall thickness using the Gibson-Ashby model with exponent 1.5, the fracture strength of constituent ZnO tends to increase with decreasing wall thickness.

### Thickness-independent fracture strength and grain size of ZnO film

**Figure 3:**
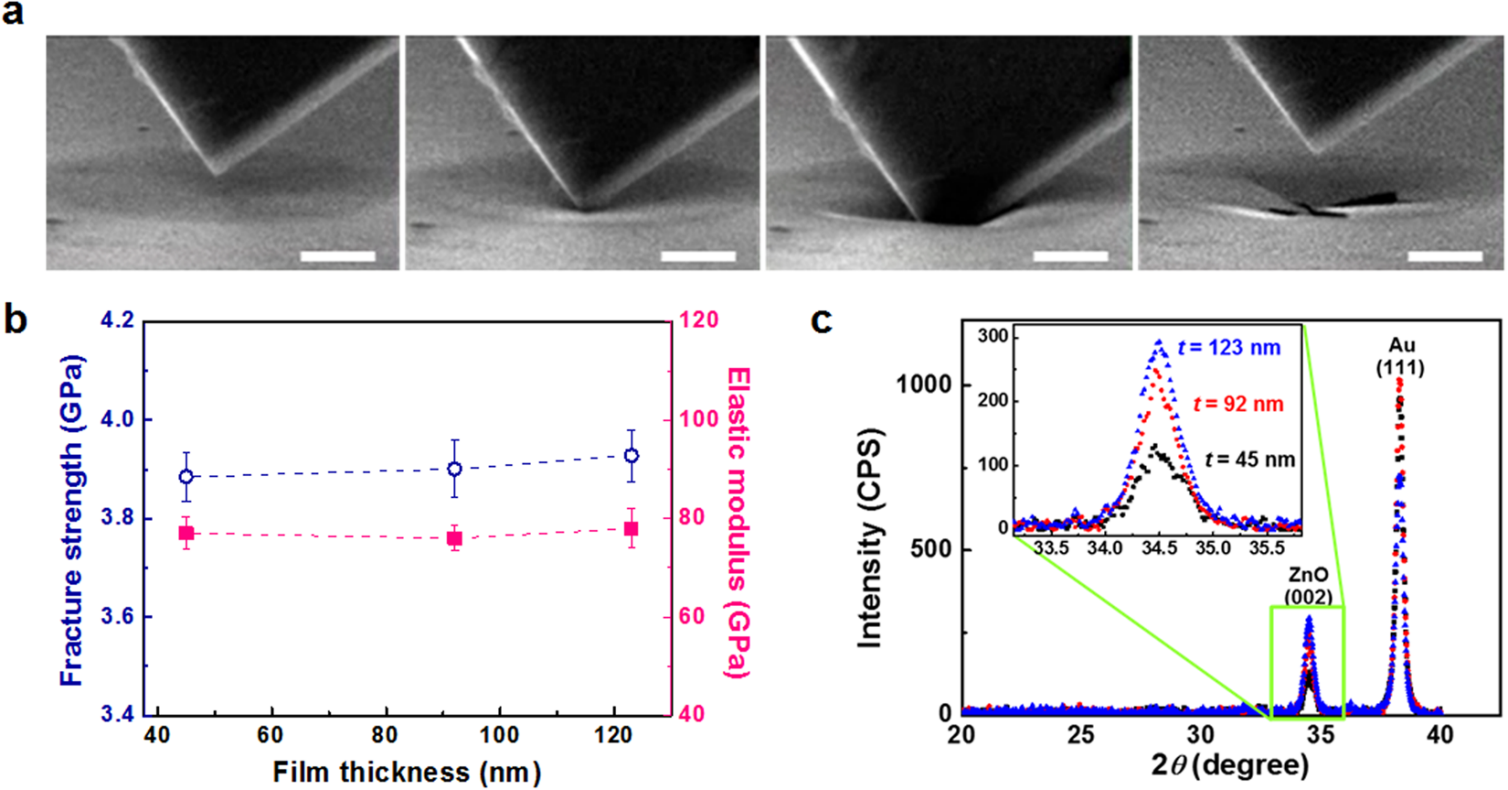
Measurement of fracture strength and grain size of ZnO film by hole-nanoindentation and grazing incidence XRD. (**a**) SEM images in *in-situ* SEM nanoindentation on suspended ZnO film (scale bars, 1 μm). (**b**) Fracture strength and elastic modulus of ZnO film is independent of film thickness. (**c**) Grazing incidence XRD reveals that ZnO film has dominant (002) texture with grain size 25 nm regardless of film thickness.

To investigate whether this apparent wall-thickness effect comes from material and/or structural properties, we performed hole-nanoindentation tests on suspended ZnO thin films (see Supplementary Movie 3) and grazing incidence XRD for ZnO thin films on Au film/Si substrates. Here, ‘material property’ means that the inherent strength of the constituent ZnO increases as wall thickness decreases, i.e. the thickness-dependent strength of ZnO^12, 26, 27^, and ‘structural property’ indicates features caused by the nanotubular structures formed on np-Au described above, based on the thickness-independent strength of ZnO. Figure 3a shows scanning electron microscope (SEM) images of hole-nanoindentation. From indentation force-displacement curves, the fracture strength and elastic modulus of ZnO films are measured using

3 $$F={{\rm{\sigma }}}_{0}({\rm{\pi }}{\rm{a}})(\frac{\delta }{a})+E({{\rm{q}}}^{3}{\rm{a}}){(\frac{\delta }{a})}^{3},$$

4 $${\sigma }_{fs}={(\frac{FE}{4{\rm{\pi }}R})}^{\frac{1}{2}},$$

where *F* and *δ* are indentation force and the displacement at center point, respectively, *σ*_0_ is the pretension in the film, *a* is the membrane radius, *q* = 1/(1.05 − 0.15*ν* − 0.16*ν* ^2^) = 0.99 with *v* = 0.2^20, 21^ is a dimensionless constant, *R* is the indenter tip radius, *E* is the elastic modulus, and *σ*_*fs*_ is the maximum stress at the central point^28, 29^. These equations are applicable to suspended membranes for which the ratios of thickness of ZnO film to hole diameter were set to be below 1%. Figure 3b shows that the fracture strength and elastic modulus of ZnO films measured by hole-nanoindentation are almost identical regardless of ZnO film thickness. The measured elastic modulus is 77 (±3.2) GPa, and fracture strength is 3.9 (±0.05) GPa, which is remarkably greater than compressive strength of sputter-deposited ZnO film reported in previous literature^18^. ZnO is a brittle material, so its strength depends on the probability that it contains flaws with high stress concentration. Jang *et al*. suggested that the fracture strength of brittle TiN shaped as hollow nanostructure increases by an exponential function with decreasing wall thickness and becomes constant theoretical strength below a critical wall thickness. The wall thicknesses of ZnO nanotubes in this study, 45, 92, and 123 nm, are likely to be below the critical wall thickness and thus the fracture strength of ZnO is likely to be independent of wall thickness.

Figure 3c shows that ZnO thin films with thicknesses of 45, 92, 123 nm have dominant (002) texture and identical grain sizes, 25 nm. Dominant (002) texture is in good agreement with previous report that ZnO synthesized by ALD has a Wurtzite structure with strong c-axis texture^30^. Grain sizes of ZnO thin films were evaluated by Debye-Scherrer equation, *D* = 0.94*λ*/*β*·cos*θ*, where *D* is the mean grain size, *λ* is the wavelength of incident X-rays (*λ* = 1.5405 Å), *β* is the full width at half maximum (FWHM) of the diffraction peak, and *θ* is the Bragg angle^31^. From hole-nanoindentation and grazing incidence XRD, we assume that wall thickness-dependent strength of nanotubular ZnO structure seen in Fig. 2d is not attributable to material properties caused by wall-thickness-dependent strength and/or microstructural change.

### Origin of apparent wall-thickness-dependent strength

**Figure 4:**
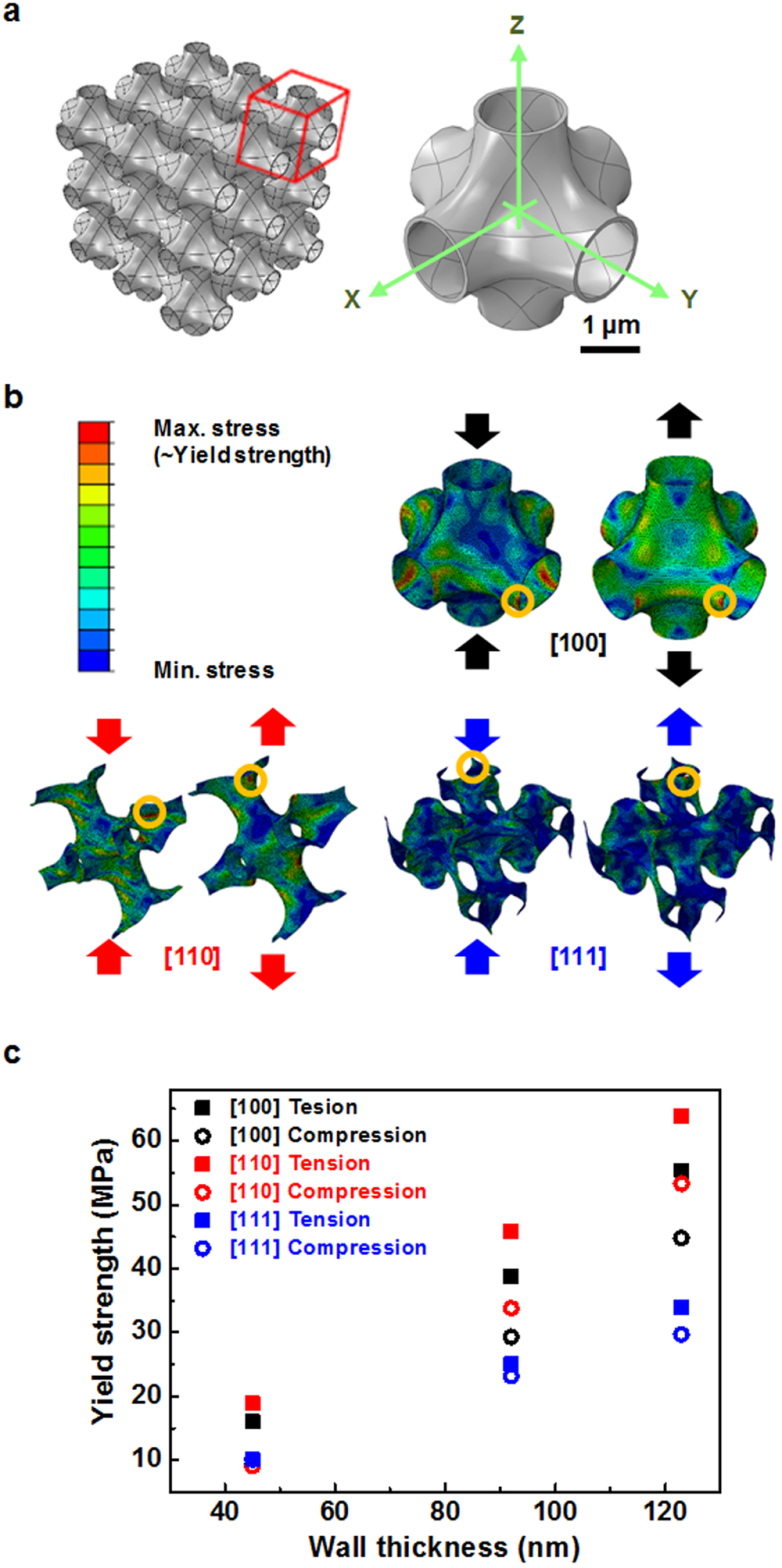
FEA simulations for nanotubular ZnO structure. (**a**) Unit cells of octahedral structure in which three tubes are interconnected normal to one another with curved surfaces are connected with periodic boundary conditions along x, y, and z directions. Representative loading directions are chosen as [100], [110], and [111]. (**b**) Distribution of von Mises stress when local von Mises stress attains a critical value in compression and tension for representative three loading directions. (**c**) Relation between yield strength of nanotubular ZnO structure evaluated by FEA simulations and wall thickness.

**Figure 5:**
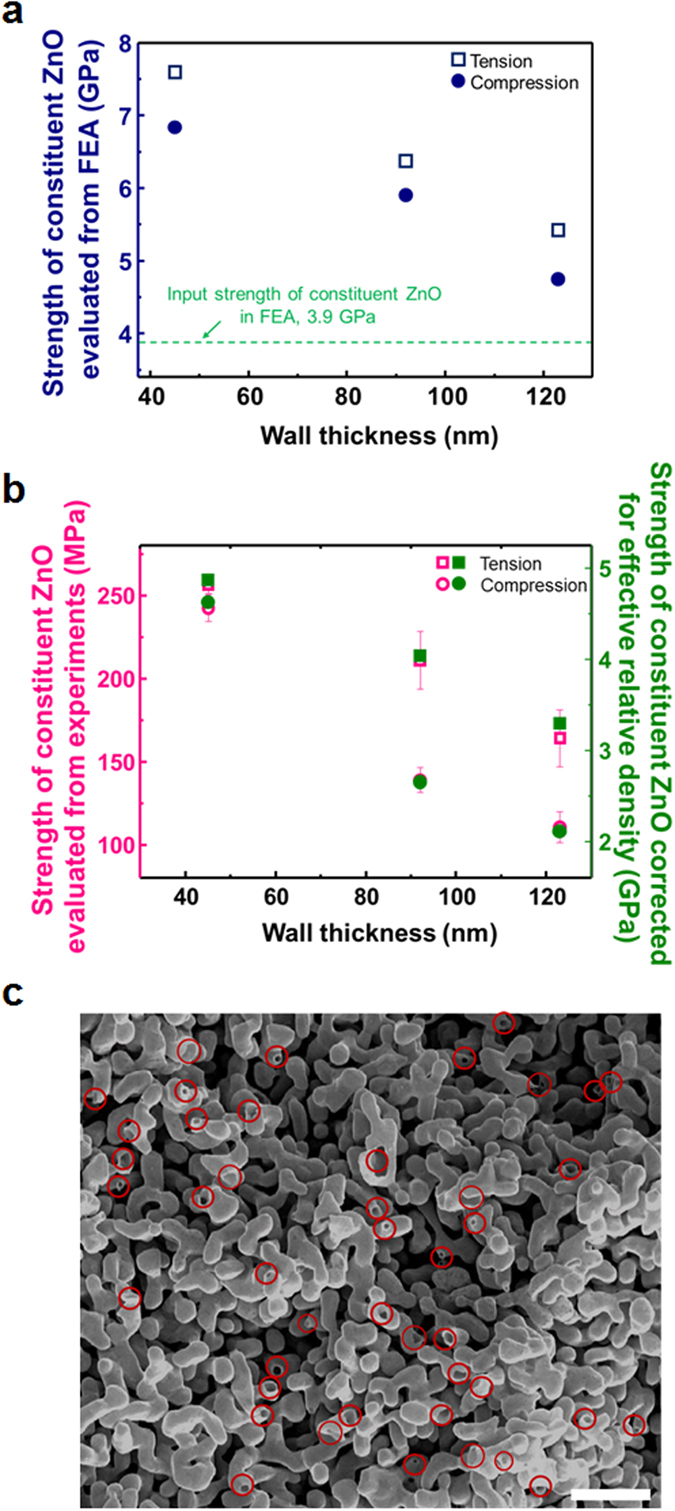
Strength of constituent ZnO evaluated by the Gibson-Ashby model as a function of wall thickness. (**a**) Strength of constituent ZnO in nanotubular structure evaluated from yield strengths evaluated by FEA simulations, by applying general Gibson-Ashby model $\bar{\sigma }=0.3{\bar{\rho }}^{1.5}$. (**b**) Strength of constituent ZnO evaluated from yield strengths measured by experiments by general Gibson-Ashby model (left y-axis), and those corrected for effective relative density as 14% of relative density in terms of volume fraction (right y-axis). (**c**) Fracture surface of nanotubular ZnO by micro-tensile testing (scale bar, 10 μm).

We performed FEA simulations as shown in Fig. 4 to investigate structural properties of nanotubular ZnO structure. FEA simulations were carried out for a linear elasticity condition, as is appropriate to investigate structural properties of nanotubular ZnO structure lacking wall-thickness-dependent strength of constituent solid. A simple octahedral unit cell structure is chosen in which three tubes are interconnected normal to one another with surface curvature so as to simulate curved tubes of nanotubular ZnO formed on np-Au ligaments with mostly saddle and convex curvatures. Three representative loading directions are chosen as [100], [110], and [111] directions on the basis of x, y, z axes as shown in Fig. 4a. The unit cell is repeated with periodic boundary conditions along the x, y, and z directions. Tensile and compressive displacement along three representative directions is applied, and when local von Mises stress attains a critical value, the nanotubular structure is assumed to yield. Locations with highest local von Mises stress are indicated as yellow circles in Fig. 4b. The evaluated yield strength of nanotubular ZnO structure is presented in Fig. 4c. Minimum values among in tension and compression along the three representative loading directions are collected in Fig. 5a, since yield strength of nanotubular ZnO structure is determined by initial local fracture. For *t* = 45 nm, tensile strength of nanotubular ZnO for loading direction [111] is the lowest and compressive strength for loading direction [110] is the lowest. For *t* = 92 nm and *t* = 123 nm, the tensile and compressive strength for loading direction [111] are the lowest. For all three loading directions, tensile strength is greater than compressive strength, as shown in Fig. 4c. Figure 2a and b also show that tensile strengths are greater than maximum compressive strengths for all three *t* = 45, 92, 123 nm. This is of interest since the nanotubular ZnO structure undergoes brittle fracture in tension, meaning that once local fracture occurs at the weakest spot, catastrophic failure develops, while its compressive behavior more resembles ductile deformation. These experimental and FEA results imply that nanotubular structure can support external force more in tension than in compression.

The Gibson-Ashby model is widely used to correlate relative strength $\bar{\sigma }$, defined by the ratio of the strength of cellular materials to that of the constituent solid, and relative density, $\bar{\rho }$, given by $\bar{\sigma }={C}_{1}{\bar{\rho }}^{1.5}$ where *C*_*1*_ is a proportional constant depending on the specific structure of a cellular material^25^. Figure 5a and b show the deduced strength of constituent ZnO in nanotubular structure calculated from yield strength of nanotubular structure measured in FEA (Fig. 4c) and experiments (Fig. 2a and b), respectively, by applying the Gibson-Ashby model. The value of *C*_*1*_ of 0.3 for open-cell foams is used for simplicity^25^. Deduced strengths of constituent ZnO calculated with yield strength of nanotubular structures evaluated by FEA, in Fig. 5a, are greater than input strength of constituent ZnO, 3.9 GPa in FEA, and tend to increase as wall thickness decreases. This indicates that the wall-thickness effect in strength of constituent ZnO seen in Fig. 5a can be attributed to the nanotubular structure (simplified in FEA) since wall thickness-independent strength of constituent ZnO were used as input value in the FEA.

Nanotubular ZnO was formed on np-Au ligaments with mostly saddle-shape surfaces, resulting in mostly negative curvature shells in the nanotubular ZnO. In nanotubular structures, the inner and the outer surface energies strongly depend on size and curvature, which can affect mechanical properties of ZnO nanotubular structures^32^. Inner surface energy increases and outer surface energy decreases as diameter of nanotubular structure decreases, and effective Young’s modulus increases as wall thickness decreases, which is critical when tube diameter is smaller than 10 nm^32^. This size-dependent surface energy might contribute to increase in deduced strength of constituent materials with decreasing wall thickness seen in Fig. 5a and b. Also, surface stress is calculated from the size-dependent surface energy by using the Shuttleworth equation and the relationship between surface energy and atomic strain^33^. This surface stress is very important and critical in understanding mechanical properties of nanostructures. Such size-dependent elasticity is significant when at least one of the dimensions of the nanostructures is smaller than 10 nm^34^. In our study, nanotubular ZnO are formed on np-Au with average ligament size of 1.43 μm and wall thicknesses are 45, 92, 123 nm, thus effects of surface energy on mechanical properties may not be remarkable.

Strength of constituent ZnO in nanotubular structure, evaluated from yield strength of nanotubular structure measured in experiments (Fig. 2d) by applying general Gibson-Ashby model $\bar{\sigma }=0.3{\bar{\rho }}^{1.5}$, also tends to increase with decreasing wall thickness, as shown in Fig. 5b. The strengths of constituent ZnO evaluated from FEA (Fig. 5a) are about 30~50 times greater than those from experiments (Fig. 5b). One possible cause for this difference is a loss of connectivity in np-Au, as appears on the fracture surface of nanotubular ZnO of *t* = 92 nm by micro-tensile testing in Fig. 5c. About 14% of the tubes are broken (red circles in Fig. 5b) when comparing with all tubes are interconnected periodically along x, y, and z directions in the FEA simulations. Recently, Ling *et al*. introduced a concept of effective relative density, ${\bar{\rho }}_{eff}$ in np-Au which is much lower than relative density in terms of volume fraction because np-Au contains many broken or dangling ligaments that do not support external force^35, 36^. They showed strength of constituent ligament is described accurately when strength of np-Au is analyzed by the Gibson-Ashby model combining with the effective relative density. Following this method taking into account effective relative density, we evaluated strength of constituent ZnO corrected for effective relative density, where effective relative density is assumed to be 14% of relative density in terms of volume fraction. These values are presented on the basis of right y-axis in Fig. 5b, which become much closer to those from FEA (Fig. 5a) by introducing concept of effective relative density. This means loss of connectivity is one primary cause for underestimate in strength of constituent ZnO evaluated by general Gibson-Ashby model (left y-axis in Fig. 5b). However, strengths of constituent ZnO corrected for effective relative density are still lower than those evaluated from FEA, which could be due to inaccurate approximation of effective relative density, and irregular nanotubular structure^23, 24^.

## Conclusions

We fabricated nanotubular ZnO by ALD coating on np-Au as template with wall thickness of 45, 92, 123 nm corresponding to relative density of 1.4, 2.8, 3.5%, respectively. In this range of wall thickness-to-tube diameter ratio, transition in deformation mode was not observed, but the samples showed similar deformation behavior in tension and compression. In tension, nanotubular ZnO structure showed linear elastic and brittle fracture; in compression, four stages appear as strain increases: (i) linear elastic deformation, (ii) nonlinear increase in stress up to highest stress, (iii) decrease in stress, and (iv) wavy plateau. Despite brittle deformation in tension, tensile strengths are greater than compressive strengths. Exponents in $\bar{\sigma }\propto {\bar{\rho }}^{m}$ are 1.09 for tension and 0.63 for compression. These both are lower than the 1.5 in the Gibson-Ashby model, indicating that the strength of constituent ZnO increases with decreasing wall thickness. Hole-nanoindentation and grazing incidence XRD revealed that strength and microstructure of ZnO film are independent of film thickness. Based on FEA, we suggested these mechanical behaviors of nanotubular ZnO structure is caused by nanotubular structure formed on np-Au. Mechanical properties of nanotubular materials formed on np-Au are distinct from those of three-dimensional architectures, being composed of long, straight and hollow struts. Nanotubular materials formed on np-Au are expected to be used widely by exploiting their unique mechanical behavior as well as high strength-to-weight ratio, surface area-to-volume ratio, and flexibility.

## Methods

### Fabrication of nanotubular ZnO

Np-Au was used as the sacrificial template for nanotubular ZnO. Au_30_Ag_70_ in at.% precursor alloys were made by arc melting under N_2_ environment from pure Au (99.99%) and Ag (99.99%) pellets, and homogenization was carried out under N_2_ environment at 800 °C for 72 hours. The Au_30_Ag_70_ precursor alloys were compressed to the desired disk shape using a Universal Testing Machine (Instron, Instron 5982), and both sides were gently polished using 1 μm diamond suspension. Dog-bone-shape Au_30_Ag_70_ precursor alloys for micro-tensile testing with gauge width 500 μm and gauge length 2 mm and cuboids for micro-compressive testing with dimension 0.8 mm width × 0.8 mm length × 1.6 mm height were machined using an ultra-precision nano-machine (Fanuc, Robonano α-0iβ). Machined precursor alloys were annealed at 800 °C for 24 hours to release residual stress introduced during mechanical polishing and machining. Samples were immersed in 35% nitric acid solution at 80 °C for 72 hours with stirring, producing np-Au with ligament size of 130 nm^37, 38^. Ligament sizes were measured by diameter of ligament necks, possibly thinnest part, from at least 100 measurements on SEM images. We obtained np-Au samples with average ligament size 1.43 μm shaped as micro-tensile and compressive samples by post-heat treatment at 650 °C for 6 hours.

ZnO was deposited on the np-Au template by ALD (NCD, Lucida D100) using diethylzinc (DEZ) and H_2_O as a precursor and oxidant, respectively, as shown in Fig. 1b. For ALD of ZnO on ligaments of np-Au, one cycle consists sequentially of dosing of DEZ for 0.5 s, exposure for 10 s, purging for 20 s, dosing of water for 0.2 s, exposure for 10 s, and purging for 20 s by N_2_ flow with 1600 sccm at processing temperature 150 °C. Growth rate was 1.9 Å/cycle, and we deposited ZnO for 210, 420, and 630 cycles. ZnO-coated np-Au was immersed in Au etchant to remove np-Au template selectively at 70 °C for 48 hours and rinsed with deionized water. We obtained nanotubular ZnO with three different wall thicknesses in the shapes of micro-tensile and compressive samples. We measured wall thickness of ZnO using SEM (FEI, NovaNano 230), confirming that the wall thickness of ZnO is uniform for the entire volume of samples.

### Micro-tensile and compressive testing

Micro-tensile tests for nanotubular ZnO were performed with a custom-built nano-tensile tester with load cell capacity of 500 mN and piezoactuator with displacement resolution of 1 nm. Tensile strain was analyzed by digital image correlation (DIC, VIC-2D by Correlated Solutions, Columbia, SC, USA). Compression tests for nanotubular ZnO were carried out using a micro-universal testing machine (Instron, Instron 5948).

### Hole-nanoindentation and grazing incidence XRD

To measure mechanical properties and to analyze ZnO microstructure, we performed hole-nanoindentations and grazing incidence XRD on ZnO thin films. ZnO films were ALD coated on 50 nm-thick Au thin film on Si substrate, in an ALD chamber together with np-Au templates for nanotubular ZnO. Au films were etched by Au etchant, which makes ZnO film float on the solution, and ZnO films were transferred onto hole-patterned Si substrates with hole diameters 5, 10, and 15 μm. Hole-nanoindentations on suspended ZnO films were carried out using *in-situ* nanoindenter (Hysitron PI 87) in SEM. Hole-nanoindentation was performed with cube corner tip with tip radius 90 nm (see Supplementary Fig. S2) at the center of the hole. The microstructure of ZnO thin films were measured by grazing incidence XRD (Bruker, D8 Advance) with Cu Kα as the line source.

### FEA simulations

Abaqus 6.12 was used for FEA simulations and Abaqus CAE was used to create input files. Displacement-controlled tensile and compressive testing were carried out. Wall thicknesses of 45, 92, 123 nm and inner diameter of tube necks of 1.43 μm were used. FEA simulations for linear elasticity with elastic modulus 77 GPa and fracture strength 3.9 GPa that are measured by hole-nanoindentation and Poisson’s ratio of 0.2 was simulated. Models were chosen as three-dimensional deformable solids; their elements are presented in Supplementary Table 1.

## Electronic supplementary material

Supplementary Information

Supplementary Movie 1

Supplementary Movie 2

Supplementary Movie 3
