# Supplementary material for: Wall-thickness-dependent strength of nanotubular ZnO
Source: Sci Rep. 2017 Jun 28;7:4327. doi: 10.1038/s41598-017-04696-4 (PMC5489488; doi:10.1038/s41598-017-04696-4)
Supplement: Supplementary file 1 — Supplementary Information [file 41598_2017_4696_MOESM1_ESM.doc]

**Supplementary Information**

**Wall-thickness-dependent strength of nanotubular ZnO**

Na-Ri Kang^1^, Young-Cheon Kim^1^, Hansol Jeon^1^, Seong Keun Kim^2^, Jae-il Jang^3^, Heung Nam Han^4^ & Ju-Young Kim^1,5,*^

^1^ School of Materials Science and Engineering, UNIST (Ulsan National Institute of Science and Technology), Ulsan 44919, Republic of Korea.

^2^ Center for Electronic Materials, KIST (Korea Institute of Science and Technology), Seoul 02792, Republic of Korea.

^3^ Division of Materials Science and Engineering, Hanyang University, Seoul 04763, Republic of Korea.

^4^ School of Materials Science and Engineering, Seoul National University, Seoul 08826, Republic of Korea.

^5^ KIST-UNIST Ulsan Center for Convergent Materials, UNIST, Ulsan 44919, Republic of Korea.

* Corresponding author. E-mail: [juyoung@unist.ac.kr](mailto:juyoung@unist.ac.kr) (J.-Y. Kim)

**
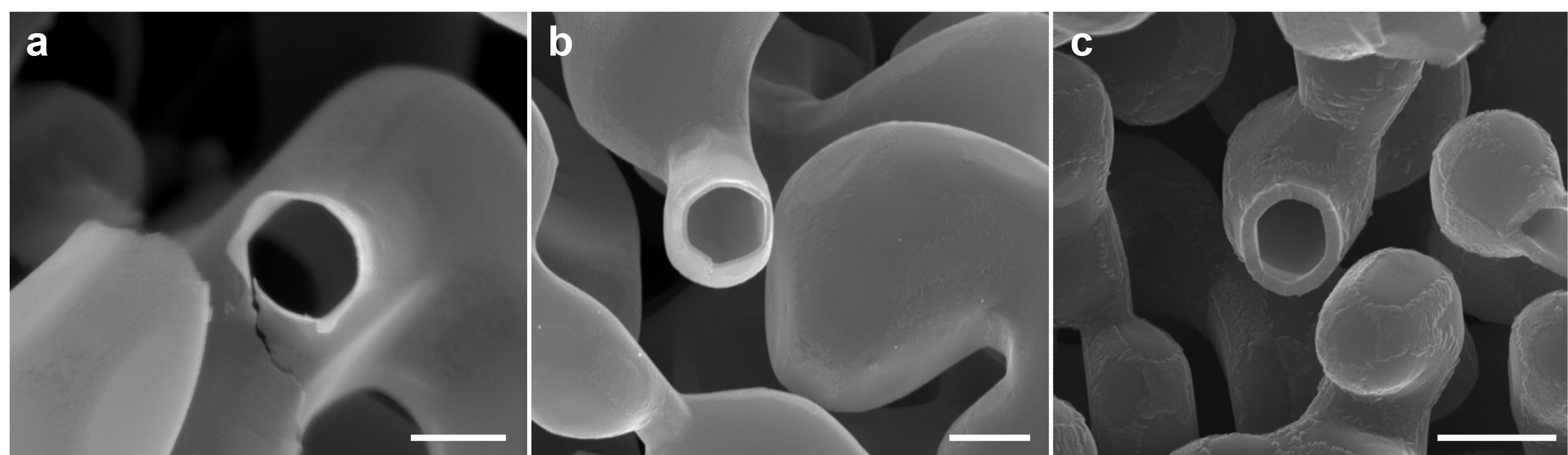
**

**Supplementary Figure 1.** SEM images of nanotubular ZnO with various wall thickness. After selective etching of np-Au template, nanotubular ZnO was cut in half and wall thickness was measured. Each wall thickness of nanotubular ZnO was measured by SEM images at least 100 measurements. (a) wall thickness *t*=45nm, (b) *t*=92nm, and (c) *t*=123nm (all scale bars, 1 μm).

**Supplementary Movie 1.** In-situ micro-tensile test video (played at 16
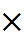
 speed) of nanotubular ZnO. Wall thickness of nanotubular ZnO is 123 nm. It shows typical brittle behavior with catastrophic failure.

**Supplementary Movie 2.** In-situ micro-compressive test video (played at 8
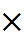
 speed) of nanotubular ZnO. Wall thickness of nanotubular ZnO is 123 nm.

**Supplementary Movie 3.** In-situ hole-nanoindentation test video (played at 16
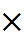
 speed) of ZnO thin film. Thickness of ZnO film is 45 nm. It shows brittle fracture with propagation of crack.


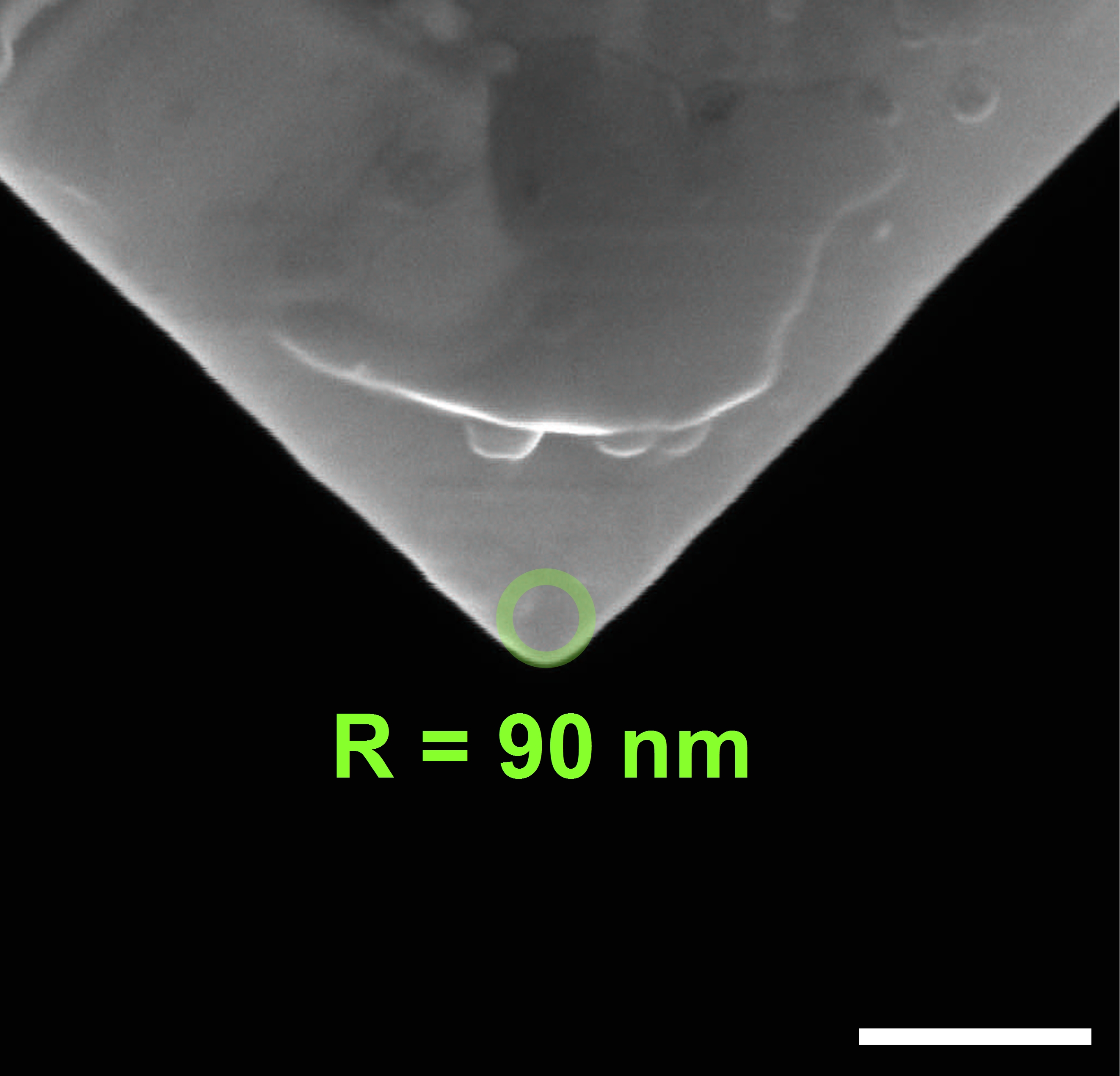


**Supplementary Figure 2.** SEM image of cube corner tip used for hole-nanoindentation. Cube corner tip with radius of 90 nm was measured by SEM according to three different angles surface (scale bar, 500 nm).

**Supplementary Table 1.** Summary of elements for FEA of nanotubular ZnO samples. The models were chosen three-dimensional deformable solid model and made up of 10-node quadratic tetrahedron elements (C3D10) according to each loading direction. Assuming that nanotubular ZnO samples are simplified to periodic array of the unit cells, symmetric boundary conditions along with x, y, and z directions were used. Displacement conditions for tensile and compression testing were applied on the top surface of unit cells. Input files were created with Abaqus CAE for 18 cases (i.e. three unit cells of three kinds of wall thickness for two loading conditions).

| **Element type** | **Wall thickness**  **(nm)** | **The number of elements** | | |
| --- | --- | --- | --- | --- |
|  |  | **Loading direction in [100]** | **Loading direction in [110]** | **Loading direction in [111]** |
| 10-node quadratic tetrahedron  (C3D10) | 45 | 51171 | 104615 | 151932 |
|  | 92 | 96439 | 209238 | 265658 |
|  | 123 | 120729 | 266822 | 320143 |
